# Anti-Inflammatory Activity of Different Agave Plants and the Compound* Cantalasaponin-1*

**DOI:** 10.3390/molecules18078136

**Published:** 2013-07-10

**Authors:** Nayeli Monterrosas-Brisson, Martha L. Arenas Ocampo, Enrique Jiménez-Ferrer, Antonio R. Jiménez-Aparicio, Alejandro Zamilpa, Manases Gonzalez-Cortazar, Jaime Tortoriello, Maribel Herrera-Ruiz

**Affiliations:** 1 Centro de Investigación Biomédica del Sur (CIBIS), Instituto Mexicano del Seguro Social (IMSS), Argentina No. 1, Col. Centro, Xochitepec 62790, Morelos, Mexico; E-Mails: monterrosasnayeli@hotmail.com (M.-B.N.); enriqueferrer_mx@yahoo.com (J.-F.E.); azamilpa_2000@yahoo.com.mx (Z.A.); gmanases@hotmail.com (G.C.M.); jtortora2@yahoo.es (T.J.); 2 Doctorado en Desarrollo de Productos Bióticos, Centro de Desarrollo de Productos Bióticos, Instituto Politécnico Nacional, P. O. Box 24, Yautepec 62730, Morelos, Mexico; E-Mails: mlarenas@ipn.mx (A.O.M.L.); arjaparicio@gmail.com (J.A.R.A.)

**Keywords:** Agavaceae, *A. angustifolia* Haw, *A. tequilana* Weber, *A. americana*, cantalasaponin-1, inflammation

## Abstract

Species of the agave genus, such as *Agave tequilana*, *Agave angustifolia* and *Agave americana* are used in Mexican traditional medicine to treat inflammation-associated conditions. These plants’ leaves contain saponin compounds which show anti-inflammatory properties in different models. The goal of this investigation was to evaluate the anti-inflammatory capacity of these plants, identify which is the most active, and isolate the active compound by a bio-directed fractionation using the ear edema induced in mice with 12-O-tetradecanoylphorbol-13-acetate (TPA) technique. A dose of 6 mg/ear of acetone extract from the three agave species induced anti-inflammatory effects, however, the one from *A. americana* proved to be the most active. Different fractions of this species showed biological activity. Finally the F5 fraction at 2.0 mg/ear induced an inhibition of 85.6%. We identified one compound in this fraction as (25*R*)-5α-spirostan-3β,6α,23α-triol-3,6-di-*O*-β-D-glucopyranoside (cantalasaponin-1) through ^1^H- and ^13^C-NMR spectral analysis and two dimensional experiments like DEPT NMR, COSY, HSQC and HMBC. This steroidal glycoside showed a dose dependent effect of up to 90% of ear edema inhibition at the highest dose of 1.5 mg/ear.

## 1. Introduction

Agaves have great economic and cultural importance for several native and cross-breed communities in Mexico, and for centuries people have used these plants as sources of food, fuel, shelter, and fiber, as fertilizer and ornamentals, but also in traditional medicine. These plant species belong to the family* Agavaceae*, of which there are some 300 species in the World, over 250 of which are found in Mexico [1].

The use of agave as food and fermented beverages source has persisted in Mexico for over 7,000 years. Tequila and mezcal, both distilled from agave, have become true Mexican symbols due to the fact that both have been certified with a “Denominación de origen” (protected designation of origin) according to the *Norma Oficial Mexicana* (NOM, Official Mexican Regulations) regulations NOM-006-SCFI-2005 (tequila) and NOM-070-SCFI-1994 (mezcal).

While these species are used as sources of fermented beverages, their use in traditional medicine has also been widely reported by Mexican herbalists [2]. *A. americana* L. is one of the 500 more widely used medicinal plants in several countries [3]. In Mexico it is used as diuretic and laxative, and also in wound, syphilis, scurvy and cancer treatments, but it’s also used to treat the lack of movement in extremities and postpartum belly inflammation [4,5,6]. Sap from *A. angustifolia* Haw is used as a treatment for digestive troubles and as a remedy for sprains and broken bones not only in people, but also in animals [7]. The main use for *Agave tequilana* (blue agave or blue weber) is the production of tequila.

The leaves of these species represent an important source of secondary metabolites like fructans, and flavonoids, but mainly terpenoids and steroidal saponins [5,8]. There are different saponins which have been isolated and identified in several species of the agave genus, such as smilagenin (steroidal saponin which precedes another eight saponins) and gitogenin [9] isolated from *A. lechugilla*. Manogenin and kamogenin, from *A. amanuensis* callus culture [10]. Hecogenin, tigogenin, agavasaponin E and H from *Agave americana* [11,12,13,14,15]. Steroidal saponins from *A. attenuata* [16,17] and *A.* *shrevei* [18]. Data in the literature indicate that agave genus species have an anti-inflammatory effect, for example, the aqueous extract of *A.* *intermixta* reduces carrageenan-induced plantar edema [19]. Steroidal saponins isolated from *A.* *attenuata* Salm-Dyck and *A.* *shevrei* Gentry, exhibit anti-inflammatory activity in a membrane permeability induced by acetic acid model [16,18]. Hecogenin, and tiogenin isolated from *A.* *americana* L., induce greater anti-inflammatory activity than the aqueous extract they come from, and even the anti-inflammatory steroidal drug, dexamethasone, in the essay of carrageenan-induced sub-plantar edema [11]. Furthermore, Mana *et al*, found that this species’ leaves have compounds with antitumor activity [20]. *A. americana* extract orally administered to sheep, with different doses of saponins (120, 240 and 360 mg/kg) has antiprotozoal activity, as well as the capacity to lower the serum concentration of cholesterol and glucose, which helps the growth of these animals’ offspring [21].

The goal of this investigation was to evaluate the anti-inflammatory effect of three species of the agave genus‒ *A.* *americana*, *A.* *tequilana* and *A.* *angustifolia—*due to the fact that there aren’t many pharmacological studies of these plants, which are widely used by several communities in Mexico. This was done using the auricular edema induced by 12-O-tetradecanoylphorbol 13-acetate (4β,9α,12β,13α,20-pentahydroxytiglia-1,6-dien-3-one 12-tetradecanoate 13-acetate, TPA) model, in addition to a chemical analysis of the species with better biological activity, which led to the structural elucidation of an anti-inflammatory compound.

## 2. Results and Discussion

**Figure 1:**
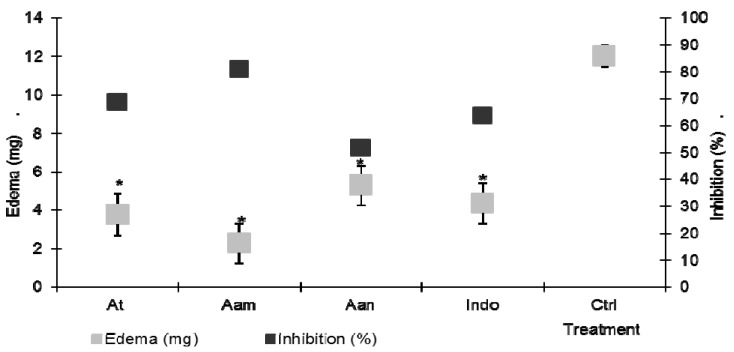
Effect of the topical administration of the acetone extract of *A. tequilana* (AT), *A. americana* (Aam), *A. angusitfolia* (Aan) at 6 mg/ear, on TPA induced ear edema. Indo = Indomethacin. ANOVA, *post-hoc* Bonferroni * *p* < 0.05 (n = 7, mean + SD, when it is compared with the negative control).

Topical administration of TPA caused an approximately 12 mg edema in this test (Figure 1, negative control group, Ctrl). The concentration employed in the present study was selected after performing some screening assays of TPA-induced auricular edema to analyze the anti-inflammatory activity of *A. americana* y *A. tequilana* and* A. angustifolia* acetone extracts. We decided to evaluate them at 1.0, 2.0, 4.0, 6.0, y 10.0 mg/ear, and significant biological activity was seen starting from 2.0 mg/ear. There were no statistical differences between groups (data not shown), but the 6.0 mg/ear effect was higher, so we chose that concentration to perform our assays. It was observed that the groups that received *A.* *tequilana* (At), *A.* *americana* (Aam) and *A.* *angustifolia* (Aan) showed a significant decrease (*p*< 0.05) of edema caused by TPA, having inflammation levels of only 3.7 mg, 2.2 mg and 5.7 mg, corresponding to inhibition percentages of 68%, 81% and 51%, respectively. Mice that received indomethacin locally (indo to 1.0 mg/ear, positive control) showed an average level of 4.3 mg (67.2%) of edema inhibition, which was significantly different from control group (*p* < 0.05, Figure 1. The aqueous extract of *Agave intermixta* was previously tested using the TPA model and showed an edema inhibition percentage of 54.27 and 56.55% with doses of 3 mg/ear and 5 mg/ear, respectively [19].

So far, in the literature there was only one report in 1997 of any such activity from *A. americana*. In that paper the authors showed that an extract of this species (at doses of 200 and 300 mg/kg, ip) and the corresponding mixture of genins (hecogenin and tiogenin) induced a decrease in carrageenan-induced plantar edema in Wistar rats [11].

There are no reports of such activity in the species* A.* *tequilana* and *A.* *angustifolia*. As seen in Figure 1, the *A.* *americana* acetone extract showed higher activity than the other two species at the same dose, and even higher than the reference drug indomethacin. This nonsteroidal anti-inflammatory drug is widely used to treat edema due to its ability to block the inflammatory cascade associated with arachidonic acid by inhibiting the enzymes cyclooxygenase 1 (COX1) and 2 (COX2). There are pharmacological reports which indicate the anti-inflammatory effect of different species of the agave genus, which is mainly attributed to the presence of steroidal saponins [16,18] and terpenes [22]. Thus, the anti-inflammatory activity of several species of the agave genus has already been established by several tests, including the one induced with TPA for *A.* *intermixta* Trel [19], and also in an induced vascular permeability test with acetic acid to *A.* *attenuata* Salm-Dyck [16] and *A.* *shevrei* Gentry [18].

Apparently, *A. americana* has the ability to decrease the inflammatory process caused by different substances, and their effect may be due to local or systemic application. Both carrageenan [23] and TPA [24], activate the cyclo-oxygenase pathway, in addition to being sensitive to drugs that act as antagonists of the prostaglandins and glucocorticoids synthesis pathways.

**Figure 2:**
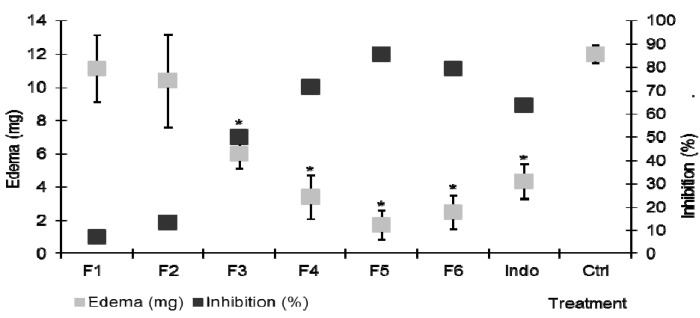
Effect of the topical administration (2 mg/ear) of the fractions with different polarity from acetone extract of *A. americana* on TPA induced ear edema. Indo = indomethacin (1 mg/ear). ANOVA, *post-hoc* Bonferroni * *p* < 0.05 (n = 7, mean ± SD, when it is compared with the negative control).

Due to its higher activity, a bio-guided chemical fractionation of *A. americana* was carried out. Six fractions with different chromatographic profile (F1 to F6, with ascending polarity) could be separated by open column chromatography (CC). Each one of these in turn was evaluated in the TPA-induced ear edema at a dose of 2 mg/ear. Lower polarity fractions F1 and F2 did not cause any significant changes (*p* > 0.05, Figure 2) in ear edema level (11.14 mg and 10.4 mg, respectively) compared to the inflammation control group (12 mg).

Ear edema, measured as a weight difference between the ears, was 6.02 mg in the group that received fraction F3, which represents a 49% inhibition (Figure 2), indicating the presence of some anti-inflammatory compounds. As the polarity of the extracts F4, F5 and F6 increased, the biological effect also increased, showing an edema level of 3.4, 1.7 and 2.4 mg, which represents a 71.7%, 85.6% and 79% edema inhibition, respectively, being significantly different from control group (*p* < 0.05, Figure 2). The data thus suggests that this plant contains more than one compound with the capacity to inhibit the local effects of TPA.

**Figure 3:**
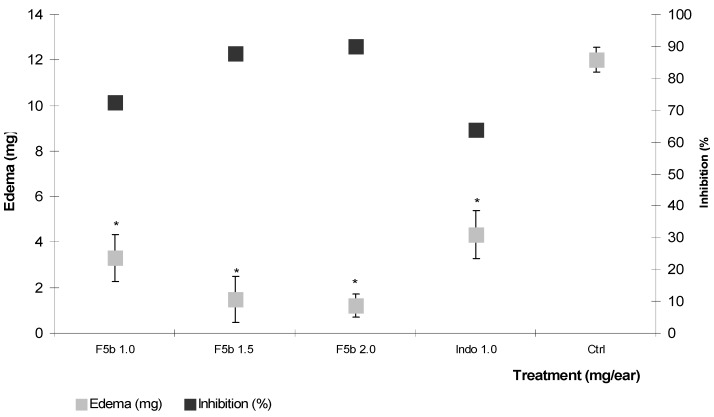
Effect of the topical administration of different doses (1.0. 1.5 and 2.0 mg/ear) of precipitate F5b isolated of *A. americana*, on TPA induced ear edema. Indo = Indomethacin (1 mg/ear). ANOVA, *post-hoc* Bonferroni, * *p* < 0.05 (n = 7, mean ± SD, when it is compared with the negative control).

Fraction F5 was separated chemically because it caused a higher edema decrease. From the fractionation of F5, a precipitate (F5b) was isolated, which showed anti-inflammatory activity in the TPA model, so it was decided to evaluate it at three doses (1.0, 1.5 and 2.0 mg/ear). The tests indicated that the three doses employed induced inflammation decreases of 3.3, 1.4 and 1.2 mg, corresponding to inhibition percentages of 72.5%, 87.6% and 90%, respectively. Although there were no statistical differences between the analyzed groups, a dose-dependent behavior was observed, and F5b had a higher anti-inflammatory effect than indomethacin at a dose of 1.0 mg/ear (Figure 3).

The precipitate F5b was exhaustively acetylated to give a white solid whose ^13^C-NMR spectroscopic analysis showed 57 signals, 29 of which could be assigned to an acetylated spirostanol skeleton type due to the characteristic ketal carbon signal at δ 108.6 (C-22). The other 28 carbon signals corresponded to the presence of two sugar moieties. The anomeric signals were observed at δ 4.62 (d, 8 Hz)/98.1 for H-1′ and C-1′ and δ 4.46 (d, 8 Hz)/101.7 for H-1′′ and C-1′′, respectively. The interglycosidic connectivities were determined by the long-range heteronuclear coupling correlations (^3^*J*_CH_) observed in the HMBC experiment of C-1′ (δ_C_ 98.1) of glucose with H-3 (δ_H_ 3.49) and C-1′′ (δ_C_ 101.7) of glucose with H-6 (δ_H_ 3.26) (Table 1). Taken together this indicated to us that the parent compound corresponded to (25*R*)-5α-spirostan-3β,6α,23α-triol-3,6-di-*O*-β-D-glucopyranoside (Table 1). This saponin has been previously described from *A. cantala* as cantalasaponin-1 [25], later it was also identified in *A. americana* [13,26].

**Table 1:** ^1^H (400 MHz) and ^13^C-NMR (100 MHz) data of the peracetylated derivative of cantalasaponin-1.

Position	δ_C_	δ_H_	Position	δ_C_	δ_H_
			3- *O*-glc		
1	39.6		1′	98.7	4.62 (d, 8)
2	27.8		2′	71.4	4.92 (dd, 8, 9.6)
3	78.6	3.499 (m)	3′	72.8	5.14 (dd, 9.2, 10)
4	28.9		4′	68.5	5.04 (dd, 9.6, 9.6)
5	49.3		5′	71.8	3.62 (dddd, 2, 4.8, 7.6, 12)
6	81.12	3.26 (m)	6′a	62.2	4.05 (dd, 2, 12)
			b		4.22 (dd, 4.8, 12)
7	37.1		6- *O-*glc		
8	33.8		1′′	101.7	4.46 (d, 8)
9	53.5		2′′	71.6	4.92 (dd, 8, 9.6)
10	36.6		3′′	73.2	5.14 (dd, 9.2, 10)
11	20.77		4′′	68.6	5.04 (dd, 9.6, 9.6)
12	39.69		5′′	71.8	3.62 (dddd, 2, 4.8, 7.6, 12)
13	41.1		6′′ a	62.3	4.05 (dd, 2, 12)
			b		4.22 (dd, 4.8, 12)
14	56.0				
15	31.8				
16	81.17	4.41 (m)			
17	61.7	0.75 (s)			
18	13.5	0.77 (s)			
19	16.2				
20	36.1				
21	14.2	0.90 (d, 6)			
22	108.6				
23	68.5	4.80 (dd, 11.6)			
24	34.1	1.46 (m)			
25	30.8				
26 a	65	3.37 (m)			
b		3.29 (m)			
27	16.5	0.78 (d, 6)			

Experiments analyzed in CDCl_3_, δ in ppm, *J* in Hz.

This saponin was found in some members of the Agavacea family and could act as a marker, as reported in 2004, from its isolation in the species *Furcraea selloa* which also belongs to that family [27]. Importantly, there is some evidence that this compound has biological activity. For example, its cytotoxic effects against JTC-26 cells which induce human cervical carcinoma has been reported [25]; the same activity was analyzed against human cells inducing HL-60 promyelocytic leukemia, but it was not active in those experiments [28]. Recently, this saponin was isolated from *A. sisalana* and it was evaluated again in a cytotoxicity test but on human MCF-7 breast cancer and NCI-H460 non-small cell lung cancer lines, and it was not active either [29]. The present report is the first to demonstrate the dose-dependent anti-inflammatory activity of cantalasaponin-1.

It has been shown that secondary metabolites from *A. americana* such as the steroidal saponins and triterpenes found in different parts of the plant, but mainly in its leaves, induced several activities. Examples of these componds are tigogenin, which has shown anticancer [30] and anti-inflammatory activity [11], besides promoting the union of nuclear factor Kappa B to DNA, which promotes the anti-proliferative effect associated to cancer [31]. Chlorogenin, another steroidal saponin, induces toxicity against HL-60 human promyelocytic leukemia cells [32], and also has anti-inflammatory activity [33]. Considering that cantalasaponin-1 is structurally linked with these active compounds (it contains one C-23 hydroxyl more than clorogenin, and two more than tigogenin) we can propose a direct relation to the biological activity observed in this study.

## 3. Experimental

### 3.1. General

12-O-Tetradecanoylphorbol 13-acetate [4β,9α,12β,13α,20-pentahydroxytiglia-1,6-dien-3-one 12-tetradecanoate 13-acetate, TPA, ≥99% purity (by TLC)] and indomethacin [Indo, ≥99% purity (by TLC)] were purchased from Sigma Chemical Co. (St. Louis, MO, USA). All NMR spectra were recorded in CDC1_3_ on a Varian INOVA-400 MHz instrument (operated at 400 MHz for ^1^H-NMR, NOESY, ^1^H-^1^H COSY, HSQC, and HMBC, and at 100 MHz for ^13^C-NMR). Chemical shifts are reported in parts per million (ppm) relative to tetramethylsilane (TMS).

### 3.2. Plant Material

The different agave leaves used were identified in the Biology Institute of the National University of Mexico (UNAM) by Dr. Abisai Josue García Mendoza as* Agave tequilana* F. A.C. Weber, *Agave angustifolia* Haw and *Agave americana* L. Marginata Hort. *A. americana* L. Marginata Hort. material was collected in the Toluca de Lerdo Municipality in the state of Mexico (19°17′29″N, 99°39′38″W); *A. tequilana* F.A.C. Weber and *A. angustifolia* Haw. were obtained from controlled cultivations in Tlaquiltenango (18°37′48′′N, 99°10′00′′W) in the state of Morelos.

### 3.3. Chemical Fractionation

The leaves were cut into pieces of approximately 10 cm in size, weighed and placed in trays for lyophilization (Heto Drywinner lyophilizer, Model DW3, Heto Holten A/S, Allerød, Denmark). Once the vegetable material was dry, it was weighed and ground (Pulvex plastic mill, D.F., Mexico), and then macerated in acetone for 72 h. The product thus obtained was filtered and the extract was concentrated under reduced pressure on a rotary evaporator (Heildolph Laborota Model 4000, Schwabach, Germany). Once the solvent was removed, the resulting *A.* *tequilana* (At), *A.* *americana* (Aam) and *A.* *angustifolia* (Aan) extracts were lyophilized and refrigerated at 4 °C until further use.

**Figure 4:**
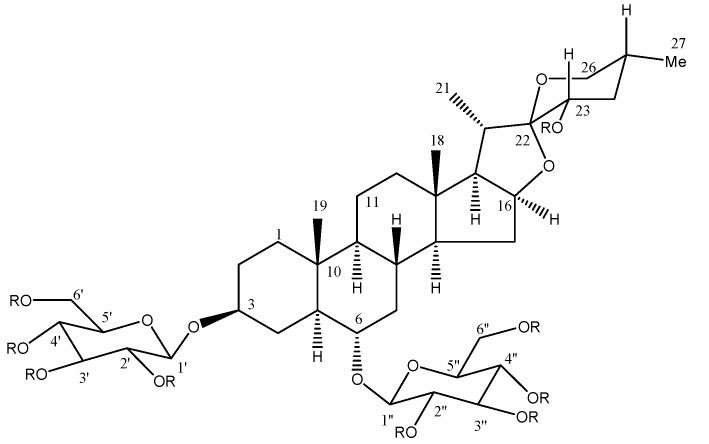
Chemical structure of cantalasaponin-1 and its peracetylated derivative.

The most biologically active extract, in this case the *A.* *americana* one, was subjected to partition with an immiscible 1:1 mixture of water/ethyl acetate (1 L, three times). The organic fraction (F-EtOAc 10 g) was concentrated by low-pressure distillation and separated on an open chromatographic column (50 × 500 mm, silica gel 70–230, 100 g, Merck, Darmstadt, Germany). The mobile phase consisted of a gradient of* n*-hexane/ethyl acetate/MeOH mixtures (250 mL each sample). Concentrated fractions (characterized by eluent composition and amount isolated) were grouped according to their chemical similarities: **F1** (100:0:0, 0.32 g), **F2** (80:20:0, 0.45 g), **F3** (50:50:0, 0.6 g), **F4** (45:45:10, 2.1 g), **F5** (40:40:20, 3.8 g) and **F6** (0:0:100, 2.3 g). Fraction **F5** afforded in acetone a white precipitate (0.5 g) which was separated by Whatman paper filtration, and denominated F5b. In order to facilitate the structure elucidation, this precipitate (100 mg) was subjected to an acetylation process (1:2 pyridine-Ac_2_O, 3 h), as previously described [34]. The crude product of this reaction was partitioned with an ethyl acetate/water mixture. The organic fraction was concentrated to dryness. Crystallization from *n*-hexane-ethyl acetate afforded the peracetate derivative (25 mg). ^1^H- and ^13^C-NMR analysis (Table 1) and two dimensional experiments like COSY, HSQC and HMBC allowed us to confirm the chemical structures shown in Figure 4.

Cantalasaponin-1 (R = H); peractylated derivative (R = COCH_3_)

### 3.4. Animals

Male ICR mice (35 gr weight) were used. All animals were purchased from Harlan Mexico (D.F, Mexico), and kept in an animal house for three weeks with a cycle of 12 h of light and 12 h of darkness and free access to water and food (pellets, Harlan). Three days before testing began the animals were conditioned to the laboratory environment and to the researcher. All experiments were conducted in accordance with the Federal Regulations for Animal Experimentation and Care (Ministry of Agriculture, NOM-062-ZOO-1999, Mexico). The experimental protocols were approved by the Research Committee of Mexican Institute of Social Security with the recording number R-2010-1701-21. The minimum number of animals and duration of observation required to obtain consistent data were employed.

### 3.5. Experimental Design

Groups of seven mice were formed, each corresponding to one of the following treatments: (a) negative control (TPA 2.5 μg dissolved in 20 µL of acetone), (b) positive control (indomethacin, 1 mg/ear) and (c) the acetone extracts of leaves of *A. tequilana*, *A. americana* and *A. angustifolia* at 6.0 mg/ear dose. The ear treatment administration procedure was initiated in the surgical anesthesia stage (pentobarbital sodium 50 mg/Kg via, ip). The left ear of each mouse served as control reference for all treatments because only 10 µL of 70% ethanol were administered on both sides of the ear. For all treatments with agave species, these were administrated 10 µL on both sides of the right ear. For positive control, the same volume of a solution of indomethacin (a non-steroidal anti-inflammatory drug) was applied and for the negative control group, only ethanol to 70%. Fifteen minutes after treatment administration, 10 µL of TPA (pro-inflammatory) solution was applied on both sides of the right ear.

Thereafter, 4 h later, the animals were sacrificed by cervical dislocation, and then 6 mm sections were taken from the ears of each mouse from all groups, the sections were weighed and the weight differential was determined [35].

In the case of the extract that showed significant anti-inflammatory effect, was performed fractionation and some selected fractions were tested (2 mg/ear). Finally, we constructed a dose-response curve for the isolated compound at the doses of 1.0, 1.5 and 2.0 mg/ear. The results were used to determine the TPA induced edema inhibition percentage was obtained using expression below:

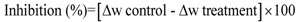

where Δw = wt − wnt; wt is the weight of the section of the treated ear; wnt is the weight of the section of the non-treated ear.

## 4. Conclusions

In this work, the anti-inflammatory activity of acetone extract of *A. angustifolia* Haw and *A. tequilana* Weber was reported for the first time. Moreover, although the anti-inflammatory effect of *A. americana* had already been described, it was previously attributed to a mixture of sapogenins such as hecogenin and tiogenin, while in this paper it is shown that the anti-inflammatory effects of this plant may also be due to the presence of cantalasaponin 1.
